# AAV9-based gene therapy partially ameliorates the clinical phenotype of a mouse model of Leigh syndrome

**DOI:** 10.1038/gt.2017.53

**Published:** 2017-07-27

**Authors:** I Di Meo, S Marchet, C Lamperti, M Zeviani, C Viscomi

**Affiliations:** 1 IRCCS Foundation Neurological Institute ‘C. Besta’, Milan, Italy; 2 University of Cambridge/Medical Research Council, Mitochondrial Biology Unit, Wellcome Trust/MRC Building, Hills Road, Cambridge, CB2 0XY, UK

## Abstract

Leigh syndrome (LS) is the most common infantile mitochondrial encephalopathy. No treatment is currently available for this condition. Mice lacking *Ndufs4*, encoding NADH: ubiquinone oxidoreductase iron-sulfur protein 4 (NDUFS4) recapitulates the main findings of complex I (cI)-related LS, including severe multisystemic cI deficiency and progressive neurodegeneration. In order to develop a gene therapy approach for LS, we used here an AAV2/9 vector carrying the human *NDUFS4* coding sequence (hNDUFS4). We administered AAV2/9-*hNDUFS4* by intravenous (IV) and/or intracerebroventricular (ICV) routes to either newborn or young *Ndufs4*^*−/−*^ mice. We found that IV administration alone was only able to correct the cI deficiency in peripheral organs, whereas ICV administration partially corrected the deficiency in the brain. However, both treatments failed to improve the clinical phenotype or to prolong the lifespan of *Ndufs4*^*−/−*^ mice. In contrast, combined IV and ICV treatments resulted, along with increased cI activity, in the amelioration of the rotarod performance and in a significant prolongation of the lifespan. Our results indicate that extraneurological organs have an important role in LS pathogenesis and provide an insight into current limitations of adeno-associated virus (AAV)-mediated gene therapy in multisystem disorders. These findings warrant future investigations to develop new vectors able to efficiently target multiple organs.

## Introduction

Complex I (cI) deficiency accounts for ~30% of oxidative phosphorylation defects^1^ and causes a variety of different diseases ranging from lethal neonatal disease to adult-onset neurodegenerative disorders. Mammalian cI is composed of 44 different proteins, 7 of which are encoded by mitochondrial DNA, the other 37 being encoded by the nuclear DNA. Fourteen subunits, conserved from prokaryotes to humans, are directly involved in the redox and proton translocating enzymatic activity, whereas 30 supernumerary subunits are involved in the assembly or regulation of the complex.^2, 3, 4^

In humans, the first nuclear-encoded pathogenic mutation in cI (that is, an AAGTC duplication at position 466–470 in exon 5) was identified in the NADH: ubiquinone oxidoreductase iron-sulfur protein 4 (NDUFS4) subunit.^5^

NDUFS4 is a non-enzymatic 18 kDa nuclear-encoded subunit that, according to the most recent cI assembly model, appears to be incorporated at a relatively late stage.^2^ Mutations in the *NDUFS4* gene are associated with Leigh syndrome (LS), a fatal progressive neurodegenerative condition of childhood characterized by symmetrical necrotizing lesions of the basal ganglia, thalamus, brainstem and cerebellum. Symptoms are highly variable, but usually include psychomotor arrest or regression, hypotonia, dystonia, ataxia, abnormal ocular movements or ophthalmoplegia, lethargy, apnoeic spells and respiratory failure.^6, 7, 8, 9^ Biochemically, elevated lactate levels in the blood and cerebrospinal fluid are frequently detected.^10^ To date, *NDUFS4* mutations have been described in 22 patients from 18 families, with symptom onset between 5 days and 4 months of life.^6^

A constitutive *Ndufs4*^*−/−*^ mouse model develops a rapidly progressive encephalopathy, starting ~40 days after birth,^11, 12^ associated with severe cI deficiency and accumulation of a catalytically inactive 830 kDa cI assembly intermediate. *Ndufs4*^*−/−*^ mouse manifests specific symptoms similar to those found in the patients, including growth retardation, ataxia, hypotonia, lethargy, failure to thrive and breathing irregularities. Magnetic resonance imaging analysis reveals hyperintense lesions primarily in the external plexiform layer of the olfactory bulb, cerebellum and vestibular nucleus of the dorsal medulla. The severe encephalopathy leads to shortened life span with >90% mortality by postnatal day (P) 50.^13^

As for the majority of mitochondrial encephalomyopathies, no treatment is currently available for LS. Here we used an adeno-associated virus (AAV) vector to deliver human *NDUFS4* (hNDUFS4) in *Ndufs4*^*−/−*^ mice and test proof-of-principle feasibility and efficacy of a gene therapy strategy aimed to ameliorate the clinical and biochemical phenotype of an otherwise fatal mitochondrial encephalomyopathy.

## Results

### Systemic AAV2/9-*hNDUFS4* is widely distributed in adult mouse tissues, restores cI assembly and activity in peripheral tissues but does not ameliorate the clinical phenotype in *Ndufs4*^*−/−*^ mice

**Figure 1:**
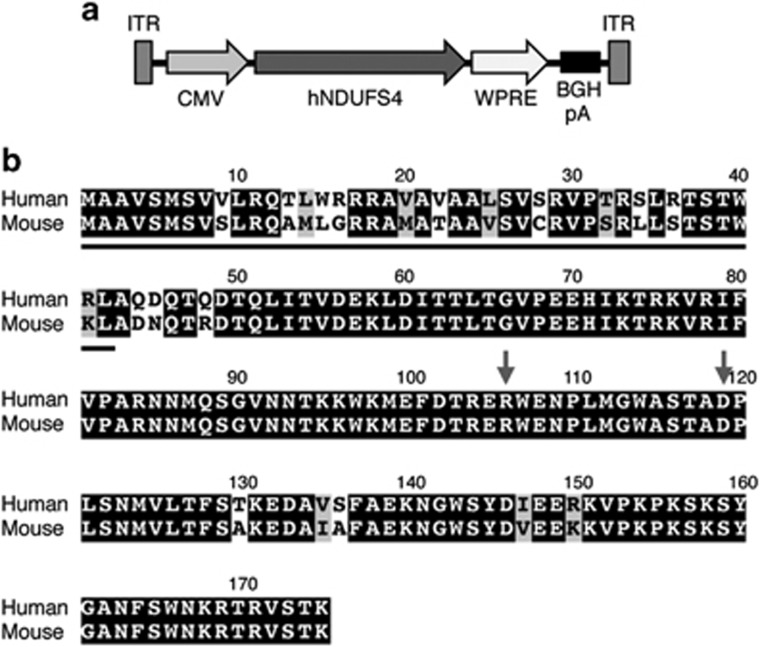
AAV2/9-CMV-hNDUFS4. (**a**) Schematic representation of AAV2/9-CMV-*hNDUFS4* vector. BGH pA, bovine growth hormone polyadenilation signal; CMV, cytomegalovirus promoter; hNDUFS4, human NDUFS4 coding sequence; ITR, inverted terminal repeats; WPRE, woodchuck hepatitis virus posttranscriptional regulatory element. (**b**) Sequence alignment of Human vs Mouse NDUFS4 proteins. The grey line indicates the predicted mitochondrial targeting sequence. Letters on black background: identities; letters on grey background: similarities; letters on white background: mismatches. Gray arrows show the position of missense mutations found in patients.

**Figure 2:**
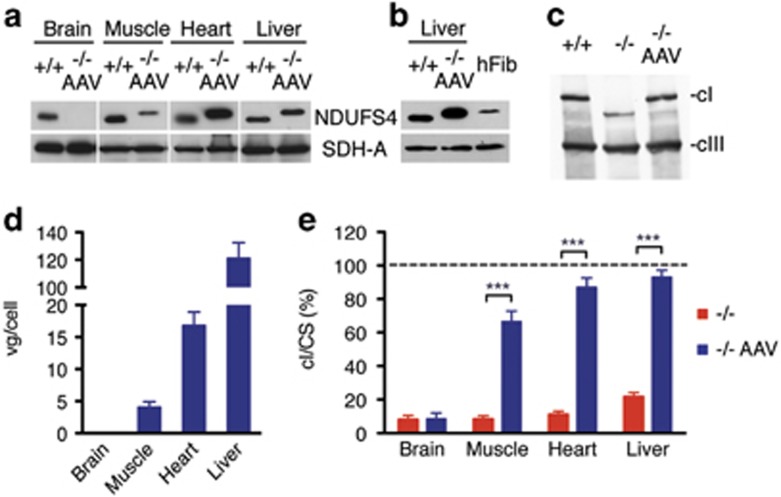
Molecular characterization of AAV2/9-CMV-hNDUFS4 IV-treated adult mice. A single retroorbital injection of 2 × 10^12^ vg was performed in *Ndufs4*^*−/−*^ mice at P21. (**a**) Western blot analysis of tissue homogenates. (**b**) Comparison of *Ndufs4*^*+/+*^ liver, *Ndufs4*^*−/−*^ AAV-treated liver and human fibroblasts (hFib) homogenates. SDH-A, the 70 kDa subunit of succinate dehydrogenase was used as protein-loading standard. (**c**) Blue-native gel electrophoresis on liver mitochondria from untreated and AAV-treated *Ndufs4*^*−/−*^ mice. (**d**) Viral genome copies (gc) content in tissues from AAV-treated *Ndufs4*^*−/−*^ mice (*n*=3). Bars indicate the s.d. (**e**) Complex I activity in tissues from *Ndufs4*^*−/−*^ (red; *n*=6) and AAV-treated *Ndufs4*^*−/−*^ mice (blue; *n*=3) normalized against *Ndufs4*^*+/+*^ mice (dashed line; *n*=6) activity, expressed as percentage of cI/citrate synthase (CS). Bars indicate s.d. The asterisks represent the significance levels calculated by unpaired, Student’s two-tailed *t*-test: *P<0.05, **P<0.01 and ***P<0.001.

In order to develop a suitable vector for gene therapy in Leigh disease, a multisystem mitochondrial disease, we cloned the human wild-type *NDUFS4* cDNA (*hNDUFS4*) into a single-stranded AAV2/9 viral vector under the control of the strong, general promoter of the cytomegalovirus (CMV) (AAV2/9-*hNDUFS4*) (Figure 1a). AAV9 has a wide tropism, which allows it to infect several tissues. This feature makes it a strong candidate for gene therapy in multisystem disorders. The hNDUFS4 protein is almost identical to the mouse (m)NDUFS4 protein (88% identity; 94% similarity), displaying 22 changes, 14 of which occur in the predicted mitochondrial targeting sequence at the N terminus (Figure 1b). We initially administered 2 × 10^12^ viral genomes (vg)/mouse intravenously (IV) by retroorbital IV injection in two *Ndufs4*^*−/−*^ mice at P21 and investigated the tissue distribution of the viral particles and the correct processing and incorporation of the mature hNDUFS4 protein into mouse cI. Western blot immunovisualization with an anti-NDUFS4 antibody showed high expression levels of hNDUFS4 protein in skeletal muscle (gastrocnemius), heart and liver, but not in the brain (Figure 2a), suggesting that AAV2/9 vector was unable to cross the adult brain–blood barrier after IV injection. Interestingly, we observed a difference in the apparent molecular weight between the endogenous mNDUFS4 and the transduced hNDUFS4 proteins, as previously reported.^14^ Comparison of the antibody-detected bands in homogenates from *Ndufs4*^*+/+*^, AAV-transduced *Ndufs4*^*−/−*^ mouse livers and human control fibroblasts confirmed that the human and mouse proteins had different molecular weights (Figure 2b). However, AAV-transduced hNDUFS4 was able to fully restore cI assembly in *Ndufs4*^*−/−*^ liver mitochondria (Figure 2c) and to rescue the cI spectrophotometric activity (Figure 1e) in all tissues (Figure 2d). No obvious differences in body weight and motor coordination by rotarod test were observed in treated vs untreated *Ndufs4*^*−/−*^ littermates (*n*=3) (Supplementary Figure 1). Similarly, the survival probability was similar between treated and untreated animals (survival median: naïve *Ndufs4*^*−/−*^ 55.0 days; AAV-treated *Ndufs4*^*−/−*^ 58.0 days).

### Systemic AAV2/9-*hNDUFS4* does not ameliorate the clinical phenotype of *Ndufs4*^*−/−*^ newborn mice

**Figure 3:**
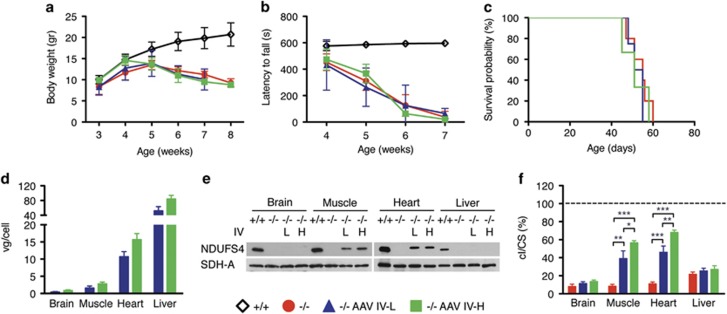
Molecular and clinical characterization of AAV2/9-CMV-hNDUFS4 IV-treated newborn mice. (**a**) Variation of body weights over time in *Ndufs4*^*+/+*^(white outline; *n*=6), *Ndufs4*^*−/−*^ (red; *n*=6), AAV IV-L-treated *Ndufs4*^*−/−*^ (blue; *n*=3) and AAV IV-H-treated *Ndufs4*^*−/−*^ (green; *n*=3) mice. Error bars represent s.d. (**b**) Rotarod analysis in *Ndufs4*^*+/+*^(white outline; *n*=6), *Ndufs4*^*−/−*^ (red; *n*=6), AAV IV-L-treated *Ndufs4*^*−/−*^ (blue; *n*=3) and AAV IV-H-treated *Ndufs4*^*−/−*^ (green; *n*=3) mice. Error bars represent SD. (**c**) Kaplan–Meier survival probability in *Ndufs4*^*+/+*^(white outline; *n*=6), *Ndufs4*^*−/−*^ (red; *n*=6), AAV IV-L-treated *Ndufs4*^*−/−*^ (blue; *n*=3) and AAV IV-H-treated *Ndufs4*^*−/−*^ (green; *n*=3). (**d**) Viral gc content in tissues from AAV IV-L-treated *Ndufs4*^*−/−*^ (blue; *n*=3) and AAV IV-H-treated *Ndufs4*^*−/−*^ (green; *n*=3). Error bars represent s.d. (**e**) Western blot analysis of tissue homogenates. SDH-A was used as protein-loading standard. (**f**) Complex I activity in tissues from *Ndufs4*^*−/−*^ (red; *n*=6) AAV IV-L-treated *Ndufs4*^*−/−*^ (blue; *n*=3) and AAV IV-H-treated *Ndufs4*^*−/−*^ (green; *n*=3) normalized against *Ndufs4*^*+/+*^ mice (dashed line; *n*=6), expressed as percentage of cI/citrate synthase (CS). Bars indicate s.d. The asterisks represent the significance levels calculated by unpaired, Student’s two-tailed *t*-test: *P<0.05, **P<0.01 and ***P<0.001.

It was previously reported that AAV2/9 can cross the brain–blood barrier when injected systemically into newborns.^15, 16^ To this end, and in order to anticipate the treatment before the onset of the neuro-muscular symptoms, we IV injected 1 × 10^12^ (IV-L) and 2 × 10^12^ (IV-H) vg per mouse in two groups of three newborn *Ndufs4*^*−/−*^ mice through the temporal vein. Both IV-L- and IV-H-injected animals showed no differences in body weight (Figure 3a) and in motor coordination (Figure 3b), compared with untreated *Ndufs4*^*−/−*^ littermates. Moreover, the two IV injections did not prolong the lifespan of treated vs untreated *Ndufs4*^*−/−*^ mice (survival median IV-L: 53.0 days; IV-H: 51.0 days) (Figure 3c). Real-time quantitative PCR analysis revealed the presence of AAV viral DNA in the brain (IV-L: 0.44±0.13 vg per cell; IV-H: 0.89±0.16 vg per cell), muscle (IV-L: 1.62±0.57; IV-H: 2.82±0.52), heart (IV-L: 10.71±1.47; IV-H: 15.72±1.71) and liver (IV-L: 52.01±11.34; IV-H: 84.28±9.51) (Figure 3d). Western blot analysis on autoptic tissues revealed expression of hNDUFS4 protein in muscle and heart of AAV-transduced *Ndufs4*^*−/−*^ mice (~50% of *Ndufs4*^*+/+*^), whereas no hNDUFS4-crossreacting material was detected in the brain and liver (Figure 3e). Although low transduction efficiency in the brain could justify the low protein levels, the lack of protein in the liver in the presence of extremely high viral DNA and hNDUFS4 mRNA (not shown) suggests a posttranscriptional silencing of the transduced gene. However, we found a remarkable correction of cI activity defect in the skeletal muscle and heart, whereas no significant effect was observed in both brain and liver. In particular, cI/citrate synthase activity in skeletal muscle was increased from 8% in *Ndufs4*^*−/−*^ mice to 39% in IV-L- and to 57% in IV-H-treated mice. Similarly, cI/citrate synthase in heart increased from 11% to 46% and 68% in IV-L and IV-H, respectively (Figure 3f).

### ICV injections of AAV2/9-hNDUFS4 slightly ameliorate the clinical phenotype in newborn *Ndufs4*^*−/−*^ mice

**Figure 4:**
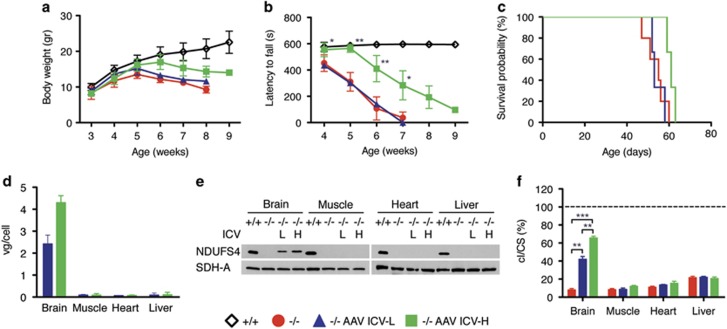
Molecular and clinical characterization of AAV2/9-CMV-hNDUFS4 ICV-treated newborn mice. (**a**) Variation of body weights over time in *Ndufs4*^*+/+*^(white outline; *n*=6), *Ndufs4*^*−/−*^ (red; *n*=6), AAV ICV-L-treated *Ndufs4*^*−/−*^ (blue; *n*=3) and AAV ICV-H-treated *Ndufs4*^*−/−*^ (green; *n*=3) mice. Error bars represent s.d. (**b**) Rotarod analysis in *Ndufs4*^*+/+*^(white outline; *n*=6), *Ndufs4*^*−/−*^ (red; *n*=6), AAV ICV-L-treated *Ndufs4*^*−/−*^ (blue; *n*=3) and AAV ICV-H-treated *Ndufs4*^*−/−*^ (green; *n*=3) mice. Error bars represent s.d. The asterisks represent the significance levels vs untreated *Ndufs4*^*−/−*^ mice calculated by unpaired, Student’s two-tailed *t*-test: *P<0.05, **P<0.01 and ***P<0.001. (**c**) Kaplan–Meier survival probability in *Ndufs4*^*+/+*^(white outline; *n*=6), *Ndufs4*^*−/−*^ (red; *n*=6), AAV ICV—–treated *Ndufs4*^*−/−*^ (blue; *n*=3) and AAV ICV-H-treated *Ndufs4*^*−/−*^ (green; *n*=3). (**d**) Viral gc content in tissues from AAV ICV-L-treated *Ndufs4*^*−/−*^ (blue; *n*=3) and AAV ICV-H-treated *Ndufs4*^*−/−*^ (green; *n*=3). Error bars represent s.d. (**e**) Western blot analysis of tissue homogenates. SDH-A was used as protein-loading standard. (**f**) Complex I activity in tissues from *Ndufs4*^*−/−*^ (red; *n*=6) AAV ICV-L-treated *Ndufs4*^*−/−*^ (blue; *n*=3) and AAV ICV-H-treated *Ndufs4*^*−/−*^ (green; *n*=3) normalized against *Ndufs4*^*+/+*^ mice (dashed line; *n*=6), expressed as percentage of cI/citrate synthase (CS). Bars indicate s.d. The asterisks represent the significance levels calculated by unpaired, Student’s two-tailed *t*-test: *P<0.05, **P<0.01 and ***P<0.001.

In order to bypass the brain–blood barrier filter, we performed intracerebroventricular (ICV) injections of AAV2/9-*hNDUFS4* using 1.5 × 10^11^ (ICV-L) and 3 × 10^11^ (ICV-H) vg per mouse in two groups of newborn (P1) *Ndufs4*^*−/−*^ mice (*n*=3). At both dosages injected animals showed no significant differences in body weights. However, ICV-H-injected mice resulted in a slight increase in body weight (Figure 4a) and in a significant improvement in motor coordination at rotarod test (Figure 4b), not present in ICV-L animals. In addition, both ICV treatments failed to prolong the lifespan of injected animals (survival median ICV-L: 55.0; ICV-H: 60 days) (Figure 4c). AAV9 DNA (ICV-L: 2.42±0.4 vg per cell; ICV-H: 4.3±0.32 vg per cell) and hNDUFS4 protein were present in the brains of all injected animals (Figures 4d and e) and, accordingly, cI activity increased from 8% in *Ndufs4*^*−/−*^ untreated animals to 42 and 65% in ICV-L- and ICV-H-treated mice, respectively (Figure 4f).

### Double IV+ICV injections in newborns of AAV2/9-*hNDUFS4* ameliorate the clinical phenotype and increases lifespan of *Ndufs4*^*−/−*^ mice

**Figure 5:**
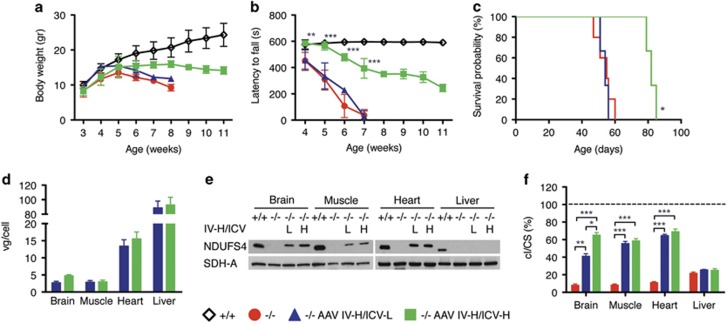
Molecular and clinical characterization of AAV2/9-CMV-hNDUFS4 IV-H/ICV-treated newborn mice. (**a**) Variation of body weights over time in *Ndufs4*^*+/+*^(white outline; *n*=6), *Ndufs4*^*−/−*^ (red; *n*=6), AAV IV-H/ICV-L-treated *Ndufs4*^*−/−*^ (blue; *n*=3) and AAV IV-H/ICV-H-treated *Ndufs4*^*−/−*^ (green; *n*=3) mice. Error bars represent s.d. (**b**) Rotarod analysis in *Ndufs4*^*+/+*^(white outline; *n*=6), *Ndufs4*^*−/−*^ (red; *n*=6), AAV IV-H/ICV-L-treated *Ndufs4*^*−/−*^ (blue; *n*=3) and AAV IV-H/ICV-H-treated *Ndufs4*^*−/−*^ (green; *n*=3) mice. Error bars represent s.d. The asterisks represent the significance levels vs untreated *Ndufs4*^*−/−*^ mice calculated by unpaired, Student’s two-tailed *t* test: *P<0.05, **P<0.01 and ***P<0.001. (**c**) Kaplan–Meier survival probability in *Ndufs4*^*+/+*^(white outline; *n*=6), *Ndufs4*^*−/−*^ (red; *n*=6), AAV IV-H/ICV-L-treated *Ndufs4*^*−/−*^ (blue; *n*=3) and AAV IV-H/ICV-H-treated *Ndufs4*^*−/−*^ (green; *n*=3). (**d**) Viral gc content in tissues from AAV IV-H/ICV-L-treated *Ndufs4*^*−/−*^ (blue; *n*=3) and AAV IV-H/ICV-H-treated *Ndufs4*^*−/−*^ (green; *n*=3). Error bars represent s.d. (**e**) Western blot analysis of tissue homogenates. SDH-A was used as protein-loading standard. (**f**) Complex I activity in tissues from *Ndufs4*^*−/−*^ (red; *n*=6) AAV IV-H/ICV-L-treated *Ndufs4*^*−/−*^ (blue; *n*=3) and AAV IV-H/ICV-H-treated *Ndufs4*^*−/−*^ (green; *n*=3) normalized against *Ndufs4*^*+/+*^ mice (dashed line; *n*=6), expressed as percentage of cI/citrate synthase (CS). Bars indicate s.d. The asterisks represent the significance levels calculated by unpaired, Student’s two-tailed *t*-test: *P<0.05, **P<0.01 and ***P<0.001.

Owing to the multisystemic phenotype of *Ndufs4*^*−/−*^ mouse model, we reasoned that IV/ICV double treatment could be more effective in ameliorating *Ndufs4*^*−/−*^ mouse phenotype. We performed IV-H injection coupled with ICV-L (IV-H/ICV-L) and ICV-H (IV-H/ICV-H) in two groups of newborn *Ndufs4*^*−/−*^ mice (*n*=3). Although IV-H/ICV-L-injected animals showed no differences in body weight and motor coordination, IV-H/ICV-H-injected animals resulted in increased body weight (Figure 5a) and in a highly significant improvement in motor coordination (Figure 5b). A moderate but significant prolongation of the survival probability was also observed (survival median IV-H/ICV-L: 53.5; IV-H/ICV-H: 82 days) (Figure 5c). Western blot analysis revealed that hNDUFS4 protein was present in the brain, muscle and heart (Figure 5e) of all IV-H/ICV-H mice, in agreement with the tissue distribution of the viral DNA (with the exception of the liver as reported above) (Figure 5d). Accordingly, cI activity was restored to wild-type levels in the muscle and heart, and to 70% of controls in the brain (Figure 5f).

**Figure 6:**
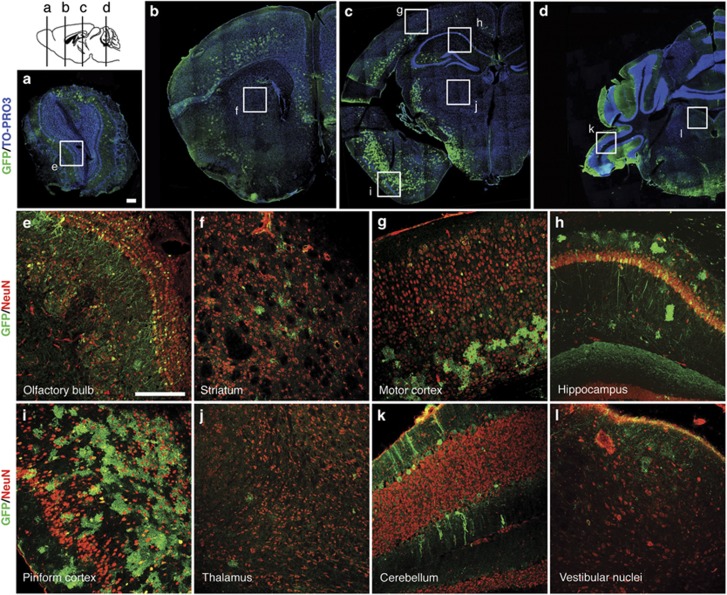
Immunofluorescence analyses on AAV2/9-CMV-eGFP ICV-treated newborn wild-type mouse brain. Newborn wild-type mouse were ICV injected with 2 × 10^10^ vg per mouse. Images showed AAV distribution at P60. (**a–d**) Double-staining with anti-GFP (green) and the nuclear marker TO-PRO3 (blue) on reconstructed brain coronal sections. (**e–l**) Double-staining with anti-GFP (green) and anti-NeuN (red) on different brain areas: (**e**) olfactory bulb, (**f**) striatum, (**g**) motor cortex, (**h**) hippocampus, (**i**) piriform cortex, (**j**) thalamus, (**k**) cerebellum, (**l**) vestibular nuclei of medulla. Scale bar: 200 μm.

Given the impossibility to detect hNDUFS4 protein by immunofluorescence, we used an AAV2/9-CMV-eGFP vector as a proxy to evaluate the distribution of the viral vector in the brain tissue. Immunofluorescence analysis of coronal brain sections from wild-type mice ICV-injected with 2 × 10^10^ vg per mouse revealed abundant green fluorescence protein (GFP) signal in several areas of the brain, including the olfactory bulb (Figure 6e), the lower layers of motor cortex (Figure 6g), the hippocampus (Figure 6h) and the Purkinje cells in the cerebellum (Figure 6k). A particularly strong signal was detected in the piriform cortex (Figure 6i). On the contrary, only few GFP-positive cells were detected in the striatum (Figure 6f), thalamus (Figure 6j) and vestibular nuclei (Figure 6l). In addition, in all areas GFP-positive cells do not colocalize with the neuron-specific marker NeuN, suggesting that glial cells are the main target of this vector.

These data suggest that the partial rescue of the phenotype is likely related to the uneven distribution of the virus in the brain parenchyma.

## Discussion

AAV-mediated gene therapy has been extensively used in pre-clinical and clinical studies for the treatment of neuromuscular disorders.^17, 18^ Here we tested the feasibility and efficacy of an AAV-based strategy on the *Ndufs4*^*−/−*^ mouse, a model of LS, a prototypical mitochondrial encephalomyopathy.

In this study, we used the *hNDUFS4* gene to rescue the murine phenotype. The human and murine proteins are highly conserved, although a slight difference of about 1 kDa in the molecular weight suggests a different cleavage, as previously reported for the bovine vs murine proteins.^14^ However, this does not impact the function since the human protein can fully rescue the cI defect in mouse liver.

The *Ndufs4*^*−/−*^ mouse model is characterized by reduced cI activity in all tissues, although a prevalent neurological component has been reported.^13^ Accordingly, systemic administration of viral particles in both adults and newborn mutant animals was unable to correct the clinical phenotype, although a marked correction of cI deficiency was observed in muscle and heart, the most affected tissues outside the CNS in early-onset cI encephalomyopathies. Interestingly, the liver was the organ with the highest viral copy number, robust expression of the recombinant transcript, but hardly any trace of Ndufs4 protein in newborn mice. However, Ndufs4 was readily detectable in animals injected at P21, suggesting the existence of a silencing mechanism restricted to the neonatal period, that inhibits hepatic translation of the recombinant gene. The elucidation of this mechanism warrants additional work in the future. Although a number of studies showed that recombinant AAV2/9 can cross the brain–blood barrier at least in newborn mice,^19^ we were unable to detect viral DNA, or the transduced mRNA/protein, in the brains of animals treated by IV injection only, either newborn or adults.

ICV administration of AAV2/9-*hNDUFS4* led to a moderate improvement of the clinical phenotype, in spite of relatively high hNDUFS4 expression levels and restoration of cI activity up to 75% of the norm. In contrast, double administration of AAV particles by both IV and ICV routes led to a remarkable improvement of the clinical signs, such as gain in body weight and amelioration in motor coordination, and highly significant prolongation of the lifespan compared with untreated *Ndufs4*^*−/−*^ mice. Palmiter and colleagues^13^ demonstrated that the *Ndufs4*^*−/−*^ model shows brain lesions mainly at level of olfactory bulbs, cerebellum and vestibular nuclei. Although the experiments with the GFP reporter showed a relatively good distribution of the virus and an improvement of cI activity in olfactory bulbs and cerebellum assessed by histochemical analysis, ICV injection of our AAV2/9 was unable to efficiently transduce vestibular nuclei, striatum and thalamus, which are critical brain structures in LS. The low transduction efficiency of basal ganglia, thalamus and striatum may explain the progressive, albeit slower, neurological disease course observed in treated mice. Notably, the GFP-based distribution of AAV2/9 in the brain seems to be restricted or predominant to glial cells, suggesting that correction of oxidative phosphorylation defects in these cell types has a protective effect on neurological functions and possibly on neuronal survival as well. This view is concordant with previous observations^20^ that the selective deletion of Tfam restricted to neurons, using the (neonatal) CamKII promoter, which is not active in glial cells, is associated with prolonged survival for several months after birth, before catastrophic neurodegeneration ensues. The beneficial effects obtained in individuals treated with combined IV and ICV injections suggests that correction of cI defects in extraneurological organs is an important component of the overall improvement in motor performance and lifespan.

In conclusion, this study warrants further development of suitable vectors able to determine a more even and robust transduction of critical brain areas. New AAV serotypes recently isolated by capsid selection method seem to be very promising to this end.^21^

## Materials and methods

### Reagents

Antibodies (NDUFA9, SDHA, CORE1) were from Mitosciences (Cambridge, UK), NDUFS4 and GFP were from Abcam (Cambridge, UK) and NeuN was from Millipore (Vimodrone (MI), Italy).

### Animals

All procedures were approved by the Italian Ministry of Health and local ethical review, in accordance with the Italian Law D.L. 26/2014 and the EU directive 2010/63/EU. The mice were kept on a C57Bl6/129Sv mixed background, and wild-type littermates were used as controls. The animals were maintained in a temperature- and humidity-controlled animal-care facility with a 12 h light/dark cycle and free access to water and food, and were killed by cervical dislocation. Body weight was monitored twice a week.

### Vector construction, production and injection

hNDUFS4 was PCR amplified, cloned into the Pcr2.1 TOPO vector (Thermofisher Scientific, Monza, Italy), sequence verified and finally cloned into the rAAV2-CMV vector. Recombinant AAV2/9-CMV-*hNDUFS4* and AAV2/9-CMV-eGFP vector particles were generated using HEK293 cells grown in serum-free suspension conditions in shaker flasks, using proprietary methods developed at the UNC Gene Therapy Center Vector Core facility (Chapel Hill, NC, USA).^22^ AAV titer was obtained by both dot blot and quantitative PCR. In adult mice, AAV particles were administered systemically by retroorbital injection.^23^ ICV and IV neonatal injections were performed as previously described.^24^ For ICV injection, pups were injected with 2–4 μl AAV2/9–CMV–*hNDUFS4* or AAV2/9-CMV-eGFP into the lateral ventricle of each hemisphere, located 1 mm lateral to the superior sagittal sinus and 2 mm rostral to the transverse sinus, to a depth of 2 mm, using an electrophysiology glass capillary connected to a sterile syringe. For IV injections, pups were injected with 25–50 μl AAV particles into the temporal vein, using a 31-gauge, 30° beveled needle syringe.

### Rotarod analysis

A rotarod apparatus (Ugo Basile, Varese, Italy) was used to assess coordination skills. After two acclimation sessions, the mice underwent three trial sessions at least 20 min apart, using a standard acceleration protocol pre-set by the constructor.

### Immunoblotting

Mouse tissues were homogenized in 10 volumes of 10 mM potassium phosphate buffer (pH 7.4). Mitochondrial-enriched fractions were collected after centrifugation at 800 g for 10 min in the presence of protease inhibitors, and frozen and thawed three times in liquid nitrogen. Protein concentration was determined by the Lowry method. Aliquots, 70 μg each, were run through a 12% SDS-polyacrylamide gel electrophoresis and electroblotted onto a nitrocellulose membrane, which was then immunodecorated with different antibodies.

For blue native gel electrophoresis analysis, liver mitochondria isolated as previously described^25^ were resuspended in 1.5 M aminocaproic acid, 50 mM Bis-Tris-HCl pH 7 and 1.6 mg dodecylmaltoside per mg of proteins, and incubated for 5 min on ice before centrifuging at 20 0003 *g* at 4 °C. Coomassie G250 (5%) was added to the supernatant. One hundred micrograms were separated by 3–12% gradient blue native gel electrophoresis and then electroblotted on nitrocellulose membranes for immunodetection.

### Genome DNA extraction and quantitative PCR

Total DNA was extracted from frozen tissues. SYBR-GREEN-based real-time quantitative PCR Invitrogen (Monza (MB), Italy) was carried out for AAV-copy number analysis as previously described^26^ using primers specific to both human and murine *NDUFS4* genes; the *RNAseP* gene was used as a reference.

### Biochemical analysis

Tissues were snap-frozen in liquid nitrogen and homogenized in 10 mM phosphate buffer (pH 7.4). The spectrophotometric activity of cI, as well as citrate synthase, was measured as previously described.^27^

### Immunofluorescence analysis

Brains from AAV2/9-CMV-eGFP-injected animals were post-fixed in paraformaldehyde 5% for 24 h at 4 °C, crioprotected in phosphate-buffered saline containing 30% sucrose for 48 h at 4 °C and then frozen in optimal cutting temperature compound in dry ice. Twenty-micrometer-thick cryostat sections mounted on gelatinated glass slices were incubated with rabbit anti-GFP (1:1000) and mouse anti-NeuN (1:1000). After washing the sections three times with phosphate-buffered saline, they were stained with Alexa Fluor 488-conjugated goat anti-rabbit IgG (1:1000, Invitrogen, Monza (MB), Italy), Alexa Fluor 568-conjugated goat anti-mouse (1:1000, Invitrogen) and TO-PRO-3 nucleic acid stain (1:1000, ThermoFisher, Monza (MB), Italy). Finally, the sections were examined using a TCS-SP8 laser confocal microscope (Leica, Buccinasco (MI), Italy).

### Statistical analysis

All numerical data are expressed as mean±s.d. Student’s unpaired two-tail *t*-test and Kaplan–Meier distribution were used for statistical analysis. Differences were considered statistically significant for *P*<0.05.

## References

[bib1] Kirby DM, Crawford M, Cleary MA, Dahl HH, Dennett X, Thorburn DR. Respiratory chain complex I deficiency: an underdiagnosed energy generation disorder. Neurology 1999; 52: 1255–1264. 10214753 10.1212/wnl.52.6.1255

[bib2] Vinothkumar KR, Zhu J, Hirst J. Architecture of mammalian respiratory complex I. Nature 2014; 515: 80–84. 25209663 10.1038/nature13686 PMC4224586

[bib3] Fiedorczuk K, Letts JA, Degliesposti G, Kaszuba K, Skehel M, Sazanov LA. Atomic structure of the entire mammalian mitochondrial complex I. Nature 2016; 538: 406–410. 27595392 10.1038/nature19794 PMC5164932

[bib4] Zhu J, Vinothkumar KR, Hirst J. Structure of mammalian respiratory complex I. Nature 2016; 536: 354–358. 27509854 10.1038/nature19095 PMC5027920

[bib5] Loeffen J, Smeitink J, Triepels R, Smeets R, Schuelke M, Sengers R et al. The first nuclear-encoded complex I mutation in a patient with Leigh syndrome. Am J Hum Genet 1998; 63: 1598–1608. 9837812 10.1086/302154 PMC1377631

[bib6] Ortigoza-Escobar JD, Oyarzabal A, Montero R, Artuch R, Jou C, Jiménez C et al. Ndufs4 related Leigh syndrome: a case report and review of the literature. Mitochondrion 2016; 28: 73–78. 27079373 10.1016/j.mito.2016.04.001

[bib7] Budde SMS, van den Heuvel LPWJ, Smeets RJP, Skladal D, Mayr JA, Boelen C et al. Clinical heterogeneity in patients with mutations in the NDUFS4 gene of mitochondrial complex I. J Inherit Metab Dis 2003; 26: 813–815. 14765537 10.1023/b:boli.0000010003.14113.af

[bib8] Petruzzella V, Vergari R, Puzziferri I, Boffoli D, Lamantea E, Zeviani M et al. A nonsense mutation in the NDUFS4 gene encoding the 18 kDa (AQDQ) subunit of complex I abolishes assembly and activity of the complex in a patient with Leigh-like syndrome. Hum Mol Genet 2001; 10: 529–535. 11181577 10.1093/hmg/10.5.529

[bib9] Rahman J, Noronha A, Thiele I, Rahman S. Leigh map: a novel computational diagnostic resource for mitochondrial disease. Ann Neurol 2017; 81: 9–16. 27977873 10.1002/ana.24835 PMC5347854

[bib10] Anderson SL, Chung WK, Frezzo J, Papp JC, Ekstein J, DiMauro S et al. A novel mutation in NDUFS4 causes Leigh syndrome in an Ashkenazi Jewish family. J Inherit Metab Dis 2008; 31 (Suppl 2): S461–S467. 19107570 10.1007/s10545-008-1049-9

[bib11] Kruse SE, Watt WC, Marcinek DJ, Kapur RP, Schenkman KA, Palmiter RD. Mice with mitochondrial complex I deficiency develop a fatal encephalomyopathy. Cell Metab 2008; 7: 312–320. 18396137 10.1016/j.cmet.2008.02.004 PMC2593686

[bib12] Calvaruso MA, Willems P, van den Brand M, Valsecchi F, Kruse S, Palmiter R et al. Mitochondrial complex III stabilizes complex I in the absence of NDUFS4 to provide partial activity. Hum Mol Genet 2012; 21: 115–120. 21965299 10.1093/hmg/ddr446

[bib13] Quintana A, Zanella S, Koch H, Kruse SE, Lee D, Ramirez JM et al. Fatal breathing dysfunction in a mouse model of Leigh syndrome. J Clin Invest 2012; 122: 2359–2368. 22653057 10.1172/JCI62923 PMC3387817

[bib14] Papa S, De Rasmo D, Scacco S, Signorile A, Technikova-Dobrova Z, Palmisano G et al. Mammalian complex I: a regulable and vulnerable pacemaker in mitochondrial respiratory function. Biochim Biophys Acta 2008; 1777: 719–728. 18455500 10.1016/j.bbabio.2008.04.005

[bib15] Miyake N, Miyake K, Yamamoto M, Hirai Y, Shimada T. Global gene transfer into the CNS across the BBB after neonatal systemic delivery of single-stranded AAV vectors. Brain Res 2011; 1389: 19–26. 21397590 10.1016/j.brainres.2011.03.014

[bib16] Rahim AA, Wong AMS, Hoefer K, Buckley SMK, Mattar CN, Cheng SH et al. Intravenous administration of AAV2/9 to the fetal and neonatal mouse leads to differential targeting of CNS cell types and extensive transduction of the nervous system. FASEB J 2011; 25: 3505–3518. 21746868 10.1096/fj.11-182311

[bib17] Ortolano S, Spuch C, Navarro C. Present and future of adeno associated virus based gene therapy approaches. Recent Pat Endocr Metab Immune Drug Discov 2012; 6: 47–66. 22264214 10.2174/187221412799015245

[bib18] Hollinger K, Chamberlain JS. Viral vector-mediated gene therapies. Curr Opin Neurol 2015; 28: 522–527. 26263476 10.1097/WCO.0000000000000241 PMC4608371

[bib19] Bourdenx M, Dutheil N, Bezard E, Dehay B. Systemic gene delivery to the central nervous system using Adeno-associated virus. Front Mol Neurosci 2014; 7: 50. 24917785 10.3389/fnmol.2014.00050 PMC4040820

[bib20] Sörensen L, Ekstrand M, Silva JP, Lindqvist E, Xu B, Rustin P et al. Late-onset corticohippocampal neurodepletion attributable to catastrophic failure of oxidative phosphorylation in MILON mice. J Neurosci 2001; 21: 8082–8090. 11588181 10.1523/JNEUROSCI.21-20-08082.2001 PMC6763882

[bib21] Deverman BE, Pravdo PL, Simpson BP, Kumar SR, Chan KY, Banerjee A et al. Cre-dependent selection yields AAV variants for widespread gene transfer to the adult brain. Nat Biotechnol 2016; 34: 204–209. 26829320 10.1038/nbt.3440 PMC5088052

[bib22] Gadalla K, Bailey M, Spike RC, Ross PD. Improved survival and reduced phenotypic severity following AAV9/MECP2 gene transfer to neonatal and juvenile male Mecp2 knockout mice. Mol Ther 2013; 21: 18–30. 23011033 10.1038/mt.2012.200 PMC3536818

[bib23] Yardeni T, Eckhaus M, Morris HD, Huizing M, Hoogstraten-Miller S. Retro-orbital injections in mice. Lab Anim (NY) 2011; 40: 155–160. 21508954 10.1038/laban0511-155 PMC3158461

[bib24] Glascock JJ, Osman EY, Coady TH, Rose FF, Shababi M, Lorson CL. Delivery of therapeutic agents through intracerebroventricular (ICV) and intravenous (IV) injection in mice. JoVE 2011; 56: e2968–e2968. 10.3791/2968 PMC3227174 21988897

[bib25] Fernandez-Vizarra E, López-Pérez MJ, Enriquez JA. Isolation of biogenetically competent mitochondria from mammalian tissues and cultured cells. Methods 2002; 26: 292–297. 12054919 10.1016/S1046-2023(02)00034-8

[bib26] Di Meo I, Auricchio A, Lamperti C, Burlina A, Viscomi C, Zeviani M. Effective AAV-mediated gene therapy in a mouse model of ethylmalonic encephalopathy. EMBO Mol Med 2012; 4: 1008–1014. 22903887 10.1002/emmm.201201433 PMC3491831

[bib27] Bugiani M, Invernizzi F, Alberio S, Briem E, Lamantea E, Carrara F et al. Clinical and molecular findings in children with complex I deficiency. Biochim Biophys Acta 2004; 1659: 136–147. 15576045 10.1016/j.bbabio.2004.09.006

