# Supplementary material for: AAV9-based gene therapy partially ameliorates the clinical phenotype of a mouse model of Leigh syndrome
Source: Gene Ther. 2017 Jul 27;24(10):661–7. doi: 10.1038/gt.2017.53 (PMC5658670; doi:10.1038/gt.2017.53)
Supplement: Supplementary Figure 1 [file gt201753x1.docx]

**
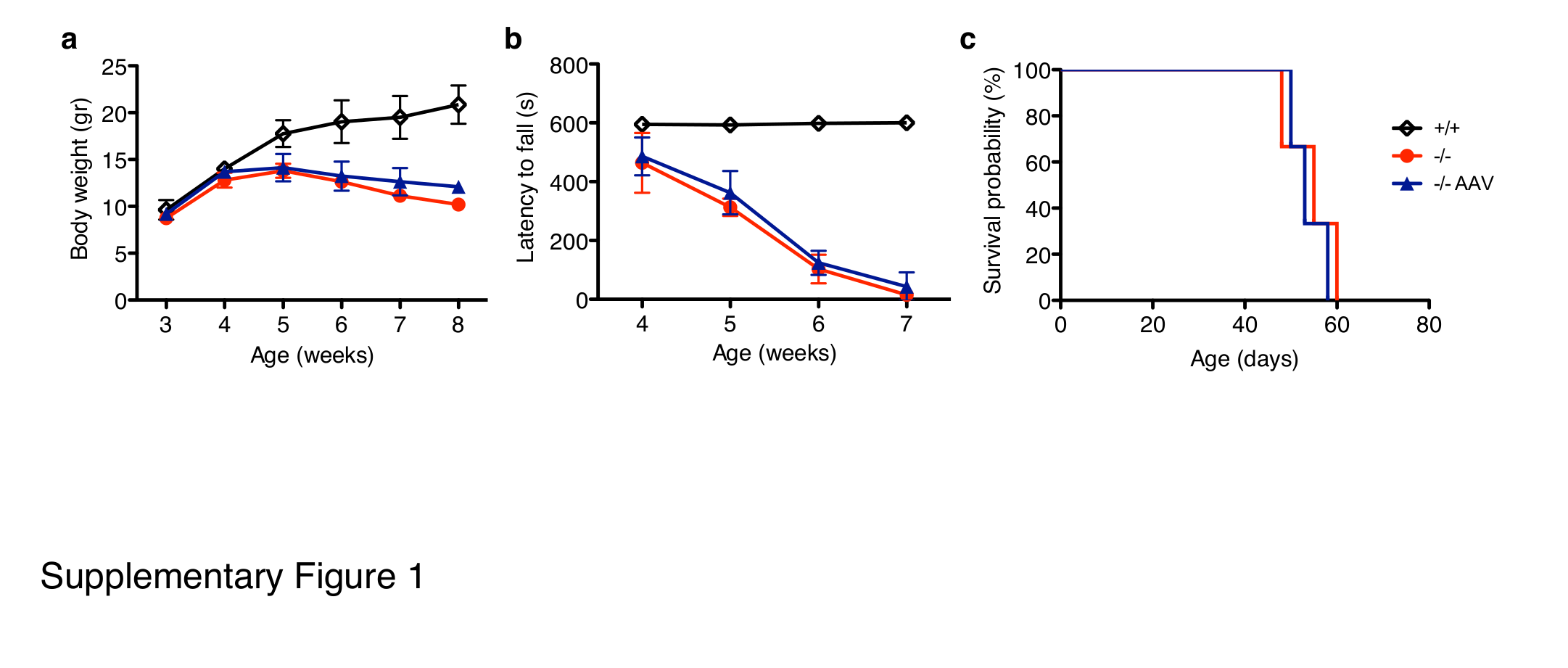
**

**
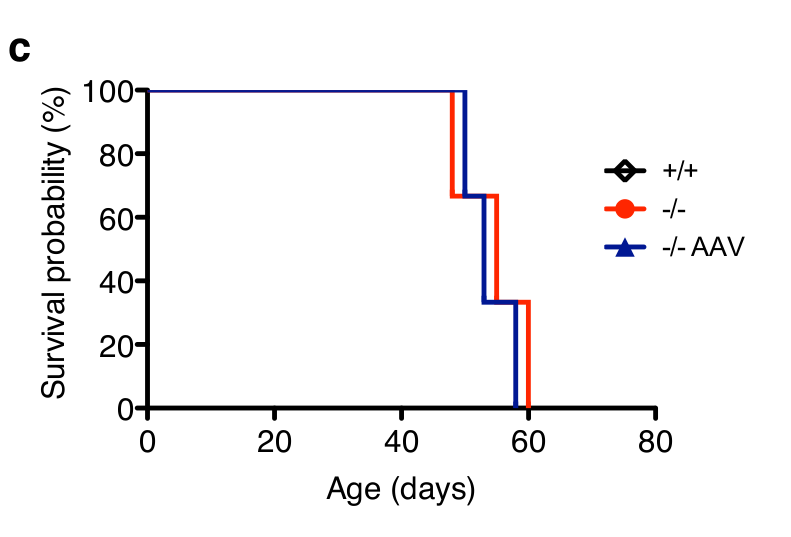
**

**Supplementary Figure 1. IV administration of AAV2/9-CMV-hNDUFS4 at P21.** (a) Body weights of injected animals. (b) Rotarod performance.

(c) Kaplan-Meier analysis of survival.

No differences were observed between AAV-treated and untreated *Ndufs4^-/-^* mice for any of the analyzed parameters.
